# Optimization of Conditions for Cryopreservation of Enriched Spermatogonial Stem Cells in Olive Flounder (*Paralichthys olivaceus*)

**DOI:** 10.3390/cells15121077

**Published:** 2026-06-13

**Authors:** Ja Young Cho, A Young Jeon, Hyun Tae Kim, Jung-Ha Kang, Jae Hun Cheong, Jae Hoon Choi

**Affiliations:** 1 Biotechnology Research Division, National Institute of Fisheries Science, Busan 46083, Republic of Korea; jjy6556@naver.com (J.Y.C.); wjsdkdud1204@gmail.com (A.Y.J.); htk8710@gmail.com (H.T.K.); genetics@korea.kr (J.-H.K.); 2 Department of Molecular Biology, Pusan National University, Busan 46241, Republic of Korea

**Keywords:** olive flounder, spermatogonia, cryopreservation, xenotransplantation

## Abstract

Spermatogonial stem cells (SSCs) are pivotal in surrogate broodstock technology. However, species-specific protocols for the efficient enrichment and long-term preservation of SSCs in olive flounder (*Paralichthys olivaceus*) are not yet fully established. In this study, we evaluated and optimized methods for the isolation and cryopreservation of *P. olivaceus* SSCs. First, we compared two enrichment methods, including Percoll density gradient centrifugation (PDGC) and differential plating (DP). Although SSCs enriched by both methods showed increased expression of SSC-specific marker genes, PDGC resulted in significantly greater enrichment than DP. A combination of PDGC and DP did not further improve enrichment efficiency, suggesting that PDGC alone is sufficient in *P. olivaceus*. Second, we optimized cryopreservation conditions according to various cryoprotectants. Among the conditions, SSCs cryopreservation using 1.3 M propylene glycol (PG) as a permeating agent and 0.2 M raffinose (Raf) as a non-permeating cryoprotectant provided the highest cell viability (56.1%), demonstrating a synergistic protective effect. Finally, preliminary in vivo migration and localization ability of the cryopreserved SSCs was confirmed through xenotransplantation into zebrafish (*Danio rerio*) larvae. PKH26-labeled donor cells exhibited successful initial localization and short-term persistence within the presumptive gonadal ridge of the recipients at 5 days post-transplantation. These findings provide an optimized protocol for the handling and preservation of *P. olivaceus* germline resources, contributing to the technical advancement of surrogate reproduction strategies in this species.

## 1. Introduction

Spermatogonial stem cells (SSCs) are responsible for the maintenance of spermatogenesis through their capacity for self-renewal and differentiation, enabling the continuous production of sperm and transmission of genetic information to subsequent generations [1,2,3]. Due to these properties, SSCs have attracted considerable attention in aquaculture biotechnology, particularly for their application in surrogate broodstock technology [4], which enables the production of donor-derived gametes through germ cell transplantation. This approach, has been recognized as a promising strategy for the conservation of genetic resources, the propagation of elite strains, and the restoration of endangered species.

In teleost fish, SSC-related technologies have advanced steadily, including the development of in vitro culture systems [2,5], germ cell transplantation [6,7], and enrichment techniques [5,8]. Enrichment of SSCs prior to transplantation is a critical step to improve colonization efficiency in recipient gonads [3]. Methods such as Percoll density gradient centrifugation (PDGC) and differential plating (DP) have been widely applied in various fish species [9]. In parallel, cryopreservation of SSCs has been considered an effective alternative to long-term in vitro culture for germline preservation. However, both enrichment efficiency and cryopreservation are highly species-specific [3,4,10], and optimized protocols are still lacking for many aquaculture species.

Olive flounder (*Paralichthys olivaceus*) is a commercially important marine fish species in East Asia, particularly in the Republic of Korea [11]. To improve its productivity, various biotechnological approaches including selective breeding [12], triploid induction [13,14], and genome editing [15] have been actively investigated. Although sperm cryopreservation techniques have been established in this species [16], the preservation of eggs or embryos remains challenging due to their large size and high lipid content [17]. Therefore, transplantation of SSCs can serve as a promising alternative to overcome these limitations, as transplanted SSCs in fish have the capacity to differentiate into either sperm or eggs depending on the recipient gonadal environment [4].

Despite their potential, studies on SSC enrichment and cryopreservation in *P. olivaceus* remain limited. In particular, comparative evaluation of enrichment methods and optimized cryopreservation conditions have not been sufficiently addressed. Therefore, the present study aimed to (i) compare the efficiency of SSC enrichment methods, including PDGC and DP, (ii) optimize cryopreservation conditions for SSCs in *P. olivaceus*, (iii) evaluate the preliminary in vivo migration and gonadal localization capability of transplanted SSCs within the presumptive gonadal ridge. Collectively, these findings provide a practical framework for SSC handling and offer a technical basis for future studies on surrogate reproduction in marine aquaculture.

## 2. Materials and Methods

### 2.1. Fish Maintenance and Ethical Statement

*P. olivaceus* used in this study were obtained from and reared at the National Institute of Fisheries Science (NIFS; Busan, Republic of Korea). The fish were accommodated in 1-ton flow-through tanks, with the water temperature regulated at 19 ± 0.3 °C under a controlled photoperiod system. A commercial extruded pellets diet (Daebong LS Co., Ltd., Jeju, Republic of Korea) was provided twice daily. All animal procedures were conducted in strict compliance with the Animal Protection Act designated by the Ministry of Agriculture, Food and Rural Affairs, Republic of Korea. The experimental protocols received formal approval from the Institutional Animal Care and Use Committee (IACUC) of NIFS (Approval No. 2025-NIFS-IACUC-20).

### 2.2. Isolation of Testicular Cells from P. olivaceus

*P. olivaceus* were anesthetized in seawater containing 0.1% (*v*/*v*) 2-phenoxyethanol (Sigma-Aldrich, St. Louis, MO, USA) for 10 min. Following anesthesia, gonadal tissues were surgically excised under sterile conditions and washed three times with 1 × Dulbecco’s phosphate-buffered saline (DPBS; Gibco, Grand Island, NY, USA) to remove residual blood and debris. The collected gonadal tissues were cut into pieces of 0.5 g and minced using a surgical blade (No. 20; Paragon, Sheffield, UK) in 100 mm culture dishes (Corning, Corning, NY, USA). The minced tissues were subjected to enzymatic dissociation using 0.25% (*v*/*v*) trypsin-EDTA (Gibco) for 2 h at 20 °C, with gentle pipetting every 10 min. After digestion, an equal volume of culture medium, consisting of high-glucose Dulbecco’s modified Eagle’s medium (DMEM; Gibco) supplemented with 20 mM HEPES (Sigma-Aldrich), 2 mM L-Glutamine (Gibco), 1 mM non-essential amino acids (Gibco), 1 mM sodium pyruvate (Gibco), 2 nM sodium selenite (Sigma-Aldrich), 100 μM 2-mercaptoethanol (Gibco), 100 U/mL penicillin-streptomycin (Gibco), 15% FBS (Gibco), 10 ng/mL bFGF, 1% fish serum, and 50 μg/mL *P. olivaceus* embryo extract (Gibco), was added to inactivate trypsin activity. The digested cells were sequentially filtered through 100 μm and 40 μm cell strainers (Falcon, Durham, NC, USA) to remove cell debris. The filtrate was centrifuged at 400× *g* for 10 min, and the cell pellet was collected for subsequent experiments.

### 2.3. Enrichment of SSCs Using PDGC and DP

To enrich the SSCs, PDGC and DP methods were used. Singular enzymatically digested total testicular cells were suspended in 300 μL of culture medium and loaded onto a discontinuous 5-layer gradient of Percoll solution consisting of 1 mL each of 20%, 30%, 40%, 50%, and 60% in 15 mL conical tubes (SPL Life Sciences, Pocheon-si, Gyeonggi-do, Korea) and centrifuged at 800× *g*, for 30 min. After centrifugation, the cells from each density fraction were harvested and washed with 1 × DPBS. Cell morphology was observed using an inverted phase-contrast microscope (Axio Vert A1; ZEISS, Göttingen, Germany). For the DP method, enzymatically digested total testicular cells were pre-plated on 0.2% (*w*/*v*) gelatin- coated 24-well plates containing cell culture medium and subsequently incubated at 20 °C for 2 days to allow preferential attachment of somatic cells. Cells that remained in suspension or were loosely attached to the substrate were carefully collected by gentle pipetting. Subsequently, the adherent cells were detached by treatment with 0.25% trypsin-EDTA (Gibco) and collected.

### 2.4. Evaluation of SSCs Enrichment Efficiency

To evaluate the enrichment efficiency of germline stem cells obtained by PDGC and DP, the expression of the SSC-specific marker genes *plzf* and *nanos2* were analyzed. Also, To compare SSC enrichment efficiency, four experimental groups were established: total testicular cells (TT) without enrichment, SSCs enriched by PDGC (PDGC), SSCs enriched by DP (DP), and SSCs enriched by sequential application of PDGC followed by DP (PDGC + DP). Total RNA was isolated using the RNeasy Micro kit (Qiagen, Hilden, Germany) following the manufacturer’s instructions. Total RNA amplification was initiated by synthesizing cDNA from 500 ng template via the SuperScript™ IV VILO™ Master Mix with ezDNase (Thermo Fisher Scientific, Waltham, MA, USA). For the subsequent qRT-PCR analysis, a QuantStudio 3 instrument (Applied Biosystems, Singapore) was employed. The reaction involved an initial step at 50 °C for 2 min and 95 °C for 10 min, followed by 40 cycles at 95 °C for 15 s and 60 °C for 30 s for gene detection. The qRT-PCR amplifications were performed in a total reaction volume of 20 µL per well, containing 250 nM of each forward and reverse primers. The primer sequences used in this study are listed in Table 1. The *18S rRNA* gene was utilized as the internal reference gene to standardize the threshold cycle (Ct) values. Relative gene expression levels were calculated using the 2^−∆∆Ct^ method.

### 2.5. Optimization of Cryopreservation Conditions for SSCs Using Different Cryoprotectants

To optimize the cryopreservation conditions for SSCs, various cryoprotectants were evaluated using extender solution consisting of sodium bicarbonate-free DMEM (Gibco) supplemented with 55.27 mM HEPES and 3.64 mM sodium pyruvate. Permeating cryoprotectants, including methanol (MeOH; Sigma-Aldrich), ethylene glycol (EG; Sigma-Aldrich), propylene glycol (PG; Sigma-Aldrich), and dimethyl sulfoxide (DMSO; Sigma-Aldrich) were individually added to the extender and evaluated by assessing post-thaw cell viability using the trypan blue dye exclusion assay to identify the most effective cryoprotectant. The optimal concentration of the selected permeating cryoprotectant was subsequently determined by testing a range of concentrations. Furthermore, non-permeating cryoprotectants including glucose (Sigma-Aldrich), lactose (Sigma-Aldrich) and raffinose (Sigma-Aldrich) were evaluated in combination with the selected permeating cryoprotectant to identify the most effective combination, and their optimal concentrations were also determined using the same protocol.

The optimization process was conducted in two sequential stages. First, to select the most effective permeating agent, methanol (MeOH), ethylene glycol (EG), propylene glycol (PG), and dimethyl sulfoxide (DMSO) were individually added to the extender at a final concentration of 1.3 M. Upon identifying PG as the optimal agent, a concentration gradient of 1.0 M, 1.3 M, and 1.5 M PG was evaluated to determine the optimal molarity. Second, to investigate synergistic protective effects, non-permeating cryoprotectants, including glucose (Glu), lactose (Lac), and raffinose (Raf), were combined with the optimized 1.3 M PG at a preliminary concentration of 0.1 M. Following the selection of Raf as the superior non-permeating agent, its concentration was further optimized by testing a range of 0.1 M, 0.2 M, and 0.3 M in combination with 1.3 M PG.

For cryopreservation, testicular cells were prepared as follows. Testicular cells collected from all fractions obtained by PDGC, excluding the bottom erythrocyte layer, were suspended and adjusted to a density of 1 × 10^6^ cells per vial, and diluted in 500 μL of extender solution in a 1.8 mL cryovial (Thermo Scientific Nunc™, Roskilde, Denmark, 375418). Subsequently, 500 μL of cryomedia containing the respective permeating or non-permeating cryoprotectants and 20% FBS (Gibco) was added to each vial and gently mixed by pipetting.

For the freezing procedure, the cryovials were placed in an isopropanol-based passive freezing container to achieve a standardized cooling rate of −1 °C/min and incubated at −80 °C overnight. On the following day, vials were moved to a −150 °C freezer for 7 days. After one week of storage, the cells were thawed in a 30 °C water bath for 2 min 30 s. Next, cell viability was assessed using trypan blue exclusion assay and the percentage of viability expressed as the proportion of viable cells relative to the total cell count [Viability (%) = non-stained cells/(stained cells + non-stained cells) × 100].

### 2.6. PKH26 Labeling and Transplantation Assay

Cryopreserved-thawed SSCs, which had been frozen under our newly optimized protocol, were used as donor cells. For in vivo tracking following transplantation, SSCs were labeled with the PKH26 Red Fluorescent Cell Linker kit for general cell membrane labeling (PKH26, Sigma-Aldrich). Briefly, cryopreserved-thawed SSCs were suspended in Diluent C and incubated with a final PKH26 dye concentration of 2 µM for 10 min at room temperature. The staining reaction was stopped by adding an equal volume of culture medium. Subsequently, the labeled cells were washed three times with DPBS via centrifugation at 400× *g* for 5 min to remove any unbound dye before transplantation. Wild-type zebrafish (*Danio rerio*) embryos were collected immediately after fertilization and maintained at 28 °C in embryo medium supplemented with 1 ppm of methylene blue. *D. rerio* larvae at 2–3 days post-hatching were used as recipients for SSC transplantation. Prior to microinjection, the larvae were anesthetized with 0.01% 2-phenoxyethanol (Sigma-Aldrich). The anesthetized larvae were transferred to a 2.5% (*w*/*v*) agarose gel mold, and 0.15 µL of PKH26-labeled SSCs (1.64 × 10^5^ cells/µL) were microinjected into the peritoneal cavity and presumptive gonadal ridge under a Leica S6E Stereomicroscope (Leica Microsystems Co., Ltd., Wetzlar, Germany). Microinjection needles were pulled from standard borosilicate glass capillaries (1B100-4, World Precision Instruments, Inc., Sarasota, FL, USA) using a puller model P-97 (Sutter Instrument, Novato, CA, USA) with the following settings: heat, 450; pull, 10; velocity, 20; and time, 210. The needles were beveled at an angle of 40° and further sharpened to obtain an inner diameter of 7–10 µm using Narishige micro-blur (EG-4, Narishige International, Tokyo, Japan). After transplantation, the larvae were transferred to a fresh embryo medium for recovery. The primary objective of this assay was to evaluate the in vivo migration and home-range localization of the donor cells; thus, the survival and colonization behavior of the labeled SSCs within the surviving larvae were monitored daily under a microscope from 1 dpt until 5 dpt. The transplanted SSCs were observed under a fluorescence microscope (Olympus Corporation, Tokyo, Japan) equipped with a filter cube (excitation wavelength: 551 nm). Non-injected larvae maintained under identical environmental conditions were utilized as a negative control group to distinguish potential tissue autofluorescence from the true PKH26 signals.

### 2.7. Statistical Analysis

**Figure 1:**
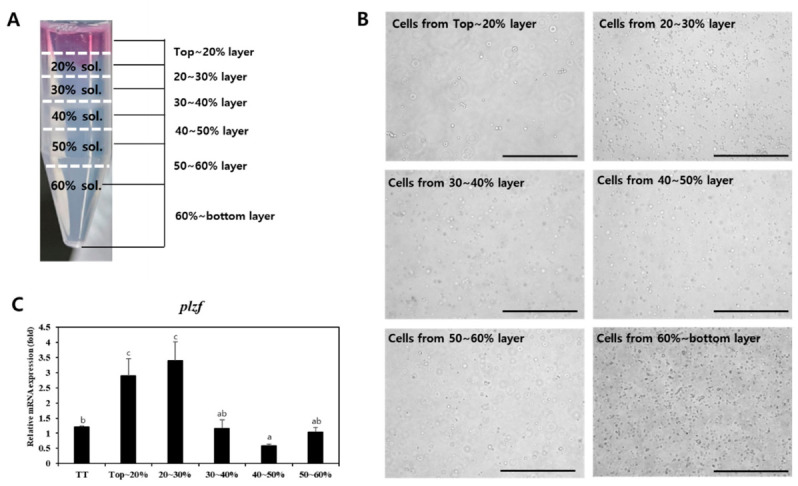
Isolation of SSCs from *P. olivaceus* testis using PDGC. (**A**) The picture of discontinuously separated testicular cells by PDGC. (**B**) Representative microscopic images of cells collected from each density fraction (Top-20%, 20–30%, 30–40%, 40–50%, 50–60%, and bottom). Scale bar = 250 μm. (**C**) Relative mRNA expression levels of the SSC marker gene *plzf* in each fraction. Data are presented as mean ± SD (*n* = 4). ^abc^ Different letters indicate significant differences among groups (*p* < 0.05). Figure 1C is reproduced from [20], Establishment and characterization of OFT and OFO cell lines from *P. olivaceus* for use as feeder cells, Biology, with permission from the publisher.

**Figure 2:**
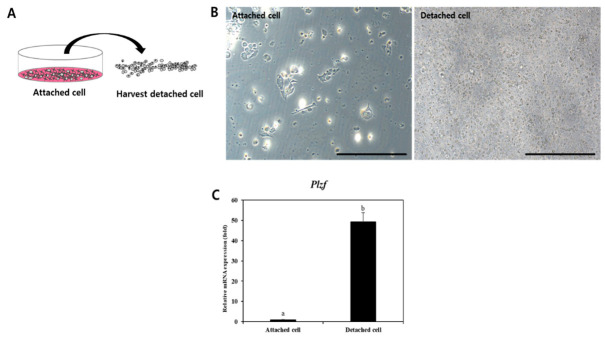
The enrichment of SSCs from *P. olivaceus* testis using DP. (**A**) Schematic illustration of the DP procedure. Testicular cells were cultured for 3 days, and weakly attached (detached) cells were collected. (**B**) Representative microscopic images of attached and detached cell populations. Scale bar = 250 μm. (**C**) Relative mRNA expression levels of *plzf* in attached and detached cells analyzed by qRT-PCR. Data are presented as mean ± SD (*n* = 3). Statistical significance was determined using a two-tailed Student’s *t*-test (*p* < 0.05). ^ab^ Different letters indicate significant differences among groups.

**Figure 3:**
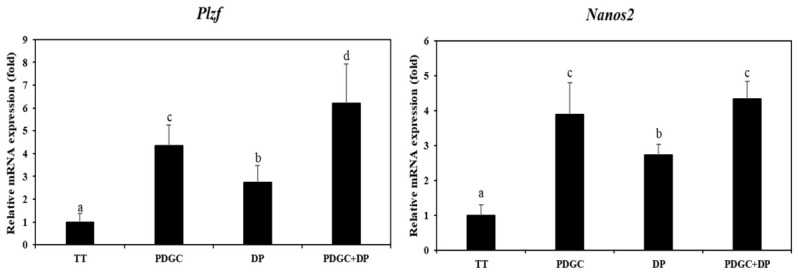
Comparison of SSC enrichment efficiency among different isolation methods. Relative mRNA expression levels of SSC marker genes (*plzf* and *nanos2*) in total testicular cells (TT), PDGC, DP, and combined PDGC + DP groups. Data are presented as mean ± SD (*n* = 3). Statistical significance was determined using one-way ANOVA followed by Duncan’s multiple range test (*p* < 0.05). ^abcd^ Different letters indicate significant differences among groups.

**Figure 4:**
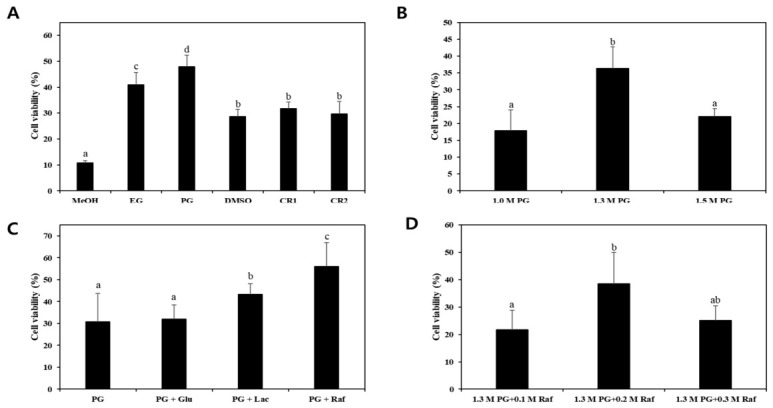
Optimization of cryoprotectant conditions for SSCs cryopreservation. (**A**) Post-thaw viability of cells cryopreserved with different permeating cryoprotectants including methanol (MeOH), ethylene glycol (EG), propylene glycol (PG), dimethyl sulfoxide (DMSO), and commercial reagents (CR1 and CR2). (**B**) Effects of different concentrations of permeating cryoprotectants on cell viability. (**C**) Post-thaw viability of cells cryopreserved with PG-based combinations containing non-permeating cryoprotectants, including glucose (Glu), lactose (Lac), and raffinose (Raf). (**D**) Effects of different concentrations of non-permeating cryoprotectants on cell viability. Data are presented as mean ± SD (*n* = 3). Statistical significance was determined using one-way ANOVA followed by Duncan’s multiple range test (*p* < 0.05). ^abcd^ Different letters indicate significant differences among groups. The screening profiles (**A**,**B**) and the optimization assays (**C**,**D**) were conducted using independent cell isolation batches, accounting for subtle baseline variations in absolute cell viability between independent experimental sets.

Data are represented as means ± standard deviation (SD). In Figure 1, Figure 2, Figure 3 and Figure 4 data were analyzed by one-way analysis of variance (ANOVA), followed by Duncan’s multiple range test using SPSS software (version 19.0; SPSS Inc., Chicago, IL, USA). Significant differences were considered when the *p* value was ≤0.05.

## 3. Results

### 3.1. Isolation of SSCs by PDGC

Following enzymatic digestion of testicular tissue, PDGC separated the cells into six discontinuous density fractions (Figure 1A). Morphological analysis of cells separated by PDGC revealed that cells in the top-20% and 20–30% fractions exhibited characteristics consistent with SSCs, including relatively small spherical shape, and a high nucleus-to-cytoplasm ratio (Figure 1B) [18]. In contrast, cells in the 60-bottom fractions displayed morphology typical of erythrocytes, such as oval or ellipsoidal nucleated shapes (Figure 1B) [19]. Consistently, gene expression analysis showed that *plzf* expression was higher in the top-20% and 20–30% fractions, with a 2.4-fold and 2.8-fold increase, respectively, compared with total testicular cells (TTs). These results indicate SSC enrichment in these fractions (Figure 1C).

### 3.2. Isolation of SSCs by DP

Following differential plating of enzymatically dissociated testicular cells, morphological observation after 3 days of culture showed that adherent cells exhibited somatic cell-like characteristics, including relatively large and heterogeneous morphology (Figure 2B).

In contrast, non-adherent cells remained in suspension and displayed features consistent with SSCs, such as smaller and round shapes (Figure 2B). Consistently, gene expression analysis revealed that *plzf* expression was higher in the non-adherent cell population, showing a 49.6-fold increase compared with the adherent cells (Figure 2C). These results indicate that the non-adherent cell population was effectively enriched for SSCs following DP.

### 3.3. Comparison of SSC Enrichment Efficiency by PDGC and DP

To compare the enrichment efficiency of SSCs, gene expression levels of *plzf* and *nanos2* were analyzed from SSCs isolated by PDGC, DP, and a combination of PDGC and DP (PDGC + DP). The expression levels of both *plzf* and *nanos2* were significantly higher in the PDGC group, showing a 4.2-fold and 4.0-fold increase compared with TT group. (Figure 3). Furthermore, the expression levels of *plzf* and *nanos2* were markedly upregulated in PDGC + DP group, with 6.4-fold and 4.4-fold elevations over the TT group (Figure 3).

The *plzf* expression levels were significantly higher in the PDGC and PDGC + DP group, showing a 1.6-fold and 2.3-fold increases compared with the DP group (Figure 3). However, no significant difference was observed between PDGC and PDGC + DP in the expression level of *nanos2*, which is a more critical marker than *plzf*. These results suggest that PDGC and PDGC + DP are more effective than DP for SSC enrichment, and that additional DP does not further improve enrichment efficiency.

### 3.4. Optimization of Cryoprotectant Conditions for SSCs Cryopreservation

To determine the optimal cryopreservation conditions for SSCs, cells were cryopreserved and subsequently thawed to evaluate the effects of different cryoprotectants on post-thaw viability. Among the permeating cryoprotectants tested, PG resulted in the highest cell viability (47.9 ± 4.5%) (Figure 4A).

Further optimization of PG concentration revealed that 1.3 M PG yielded the highest post-thaw survival (36.3 ± 2.0%) (Figure 4B). Based on this result, 1.3 M PG was subsequently combined with various non-permeating cryoprotectants. Among these combinations, PG in combination with Raf provided the highest cell viability (56.1 ± 10.8%) (Figure 4C), with the optimal concentrations identified as 1.3 M PG and 0.2 M Raf (Figure 4D). These results indicate that the combination of 1.3 M PG and 0.2 M Raf provides the optimal cryopreservation conditions for maintaining *P. olivaceus* SSC viability.

### 3.5. In Vivo Detection of Transplanted SSCs in Recipient Fish Model

**Figure 5:**
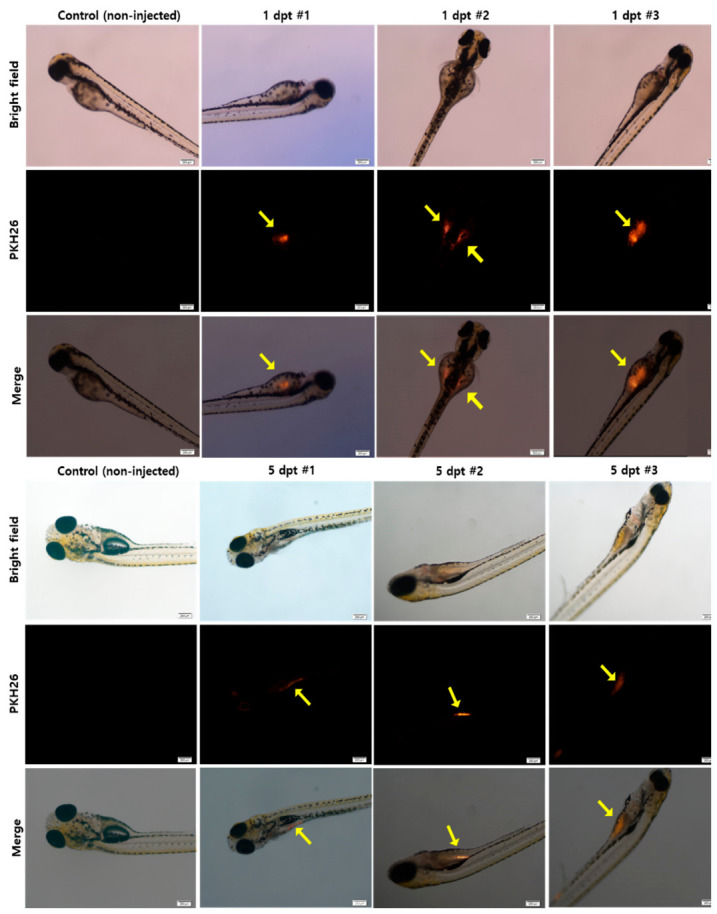
In vivo detection of transplanted *P. olivaceus* SSCs in recipient *D. rerio* larvae. PKH26-labeled donor SSCs were observed in the peritoneal cavity and the presumptive gonadal region of recipient larvae at 1 day and 5 days post-transplantation (1 dpt and 5 dpt). Fluorescence signals (PKH26) indicate donor-derived cells (yellow arrows). No fluorescence signals were detected in non-injected control larvae. Scale bar = 200 µm.

To evaluate the in vivo localization of transplanted SSCs, cryopreserved-thawed and PKH26-labeled donor cells were introduced into a recipient fish model by microinjection. PKH26-positive donor cells were clearly detected in the peritoneal cavity of recipient larvae at 1 day post-transplantation (1 dpt) (Figure 5). At 5 days post-transplantation (5 dpt), PKH26-labeled cells were detected in presumptive gonadal region, indicating the short-term persistence of transplanted cells within the recipient fish during the early post-transplantation period. In contrast, no detectable fluorescence signals were observed in the non-transplanted control larvae. These results indicate that PKH26-labeled SSCs were successfully transplanted and maintained in recipient *D. rerio* larvae at least 5 days.

## 4. Discussion

The present study optimized the conditions of enrichment and cryopreservation of SSCs in *P. olivaceus*, and observed microinjected *P. olivaceus* SSCs within the presumptive gonadal region of *D. rerio* at 5 dpt. By combining the comparison of enrichment strategies with optimization of cryoprotective conditions, this work presents a systematic optimization of enrichment and cryoprotective conditions, establishing a practical protocol for germline resource preservation in marine fish species.

Efficient enrichment of SSCs is a critical step for successful germ cell transplantation, as the proportion of undifferentiated germ cells directly affects colonization efficiency [3]. In previous studies, various approaches, including fluorescence-activated cell sorting (FACS) [8], magnetic-activated cell sorting (MACS) [21], PDGC [22], and DP [23], have been employed to enrich SSCs. However, FACS requires specialized and expensive experimental instruments, which may restrict its practical application. In addition, the application of FACS and MACS is limited in many teleost species due to the requirement for specific antibodies that reliably recognize SSC populations. To our best knowledge, no validated antibodies suitable for SSC isolation in *P. olivaceus* have been reported.

For this reason, in this study, the PDGC and DP methods were selected as accessible and practical alternatives for SSC enrichment. To systematically evaluate the enrichment efficiency of these methods, the expression levels of *plzf* and *nanos2* were selected as the specific evaluation indicators. These genes are well-established markers of SSCs in teleost fishes; in rainbow trout (*Oncorhynchus mykiss*), *nanos2* expression has been shown to be restricted to undifferentiated SSCs, while *plzf* is more broadly expressed throughout early differentiating spermatogonia for SSC maintenance and self-renewal [24].

Although no significant differences were observed between PDGC and DP, PDGC showed a significant increase in SSC marker expressions compared to total testicular cells, whereas DP was not significantly different from total testicular cells. These results indicate that PDGC, but not DP, effectively enriches SSC populations in *P. olivaceus*. Notably, the lack of additive benefit from combining PDGC with DP indicates that density-based separation alone is sufficient to resolve SSC-enriched fractions in *P. olivaceus*. This contrasts with reports in medaka (*Oryzias latipes*) [25] where the combination of PDGC and DP improves yield, suggesting that SSC isolation strategies are highly species-specific and must be empirically optimized. From a practical standpoint, the identification of PDGC as an enrichment method reduces procedural complexity and minimizes cell loss, which is particularly critical when working with limited germ cell populations.

An important step in expanding the practical application of SSCs is the establishment of reliable long-term preservation strategies. Although in vitro culture systems for SSCs have been reported in several fish species, including *O*. *latipes* [26], *D*. *rerio* [27], and *O*. *mykiss* [23], stable long-term maintenance remains challenging. In *P. olivaceus*, there is a study showing that SSCs were maintained in vitro only for 7 days [20], indicating that the in vitro culture system was not been fully established in *P. olivaceus*. In this situation, cryopreservation currently represents a feasible approach for long-term preservation of SSCs in fishes. However, optimization of conditions for cryopreservation is highly dependent on species- and cell-type-specificity [4,10]. Indeed, glycerol was identified as the most effective cryoprotectant for early-stage germ cells of the Black Sea trout (*Salmo trutta labrax*) [28], whereas EG or DMSO showed optimal results for preserving gonadal tissues of the short barbeled (*Hapalogenys nitens*) [29] and sperm of the large yellow croaker (*Larimichthys crocea*) [30]. In this context, a major contribution of the present study lies in the identification of an optimized cryopreservation condition that significantly enhances post-thaw survival rate of SSCs from *P. olivaceus*. Our results demonstrated that PG showed superior performance compared to conventional agents such as two types of commercially available reagents and DMSO. These findings are consistent with previous reports indicating that optimal cryopreservation conditions vary depending on species and cell type [4,10]. In particular, the superior performance of PG compared to various types of permeating cryoprotectants suggests that PG provides a more favorable balance of cryoprotection in *P. olivaceus* SSCs. Therefore, PG can be considered a suitable permeating cryoprotectant for the cryopreservation of SSCs in *P. olivaceus* under the conditions tested.

Importantly, the incorporation of Raf as a non-permeating cryoprotectant further improved cell viability, suggesting a potential synergistic interaction between intracellular and extracellular protective mechanisms [31]. While permeating agents mitigate intracellular ice formation [4,32], non-permeating sugars such as Raf have been hypothesized in the literature to stabilize cell membranes and help mitigate osmotic stress during freezing and thawing [32], although the precise underlying physiological pathways were not directly evaluated in the present study. The optimized combination of 1.3 M PG and 0.2 M Raf therefore potentially functions as a coordinated cryoprotective system rather than a simple additive formulation. While further studies are warranted to verify these exact cellular responses, this speculative interpretation is consistent with emerging concepts in cryobiology emphasizing the importance of multi-component protection strategies tailored to specific cell types.

Beyond cell survival, a critical question in SSC cryopreservation is whether preserved cells retain their biological functionality. In this context, the detection of transplanted SSCs within the peritoneal cavity and presumptive gonadal region of recipient *D. rerio* larvae provides important, though limited, evidence of functional competence. In fish, transplanted germ cells are known to migrate toward and localize within the gonadal region, whereas different types of cells typically exhibit ectopic localization [25,33]. Therefore, the observed initial localization of donor-derived cells in the presumptive gonadal region suggests that the transplanted SSCs retained at least fundamental biological properties. Given that early-stage colonization is a prerequisite for subsequent germline integration, these findings support the functional relevance of the optimized cryopreservation protocol.

However, it is important to acknowledge that the current study focuses on short-term post-transplantation outcomes and does not directly assess apoptosis, membrane integrity comparison between fresh and frozen-thawed cells, long-term colonization or germline transmission. While this may be viewed as a limitation, it should be interpreted in the context of the study’s primary objective, which is to establish a reproducible cryopreservation framework. Future work should extend these findings by quantitatively evaluating colonization efficiency, proliferation dynamics, and eventual germline contribution, which will be essential for fully validating the application of this system in surrogate reproduction.

Taken together, this study presents an optimized species-specific protocol for integrating SSC enrichment, cryopreservation, and initial functional validation in teleost systems. The optimized cryopreservation condition, coupled with efficient enrichment via PDGC, has direct implications for germplasm banking, selective breeding, and the development of surrogate broodstock technologies. In particular, the demonstration that cryopreserved SSCs retain the capacity for short-term in vivo persistence highlights their potential as a practical resource for germline manipulation in aquaculture.

## 5. Conclusions

In conclusion, the present study establishes a preliminary technical framework for the enrichment and cryopreservation of SSCs in *P. olivaceus*. By identifying PDGC as an effective enrichment method and optimizing a PG-raffinose-based cryopreservation protocol, this work provides foundational strategies for handling olive flounder germ cells. While further long-term studies are essential to fully validate germline transmission and the production of donor-derived offspring, our findings demonstrate the initial feasibility of SSC banking and provide a preliminary basis for the future development of surrogate reproduction technologies in marine fish species.

## Figures and Tables

**Table 1:** The information of primer sequences used in this study.

Gene		Primer Sequence (5′ > 3′)	Product Size (bp)	Accession Number
*18S rRNA*	Forward	ATGGCCGTTCTTAGTTGGTG	218	EF126037.1
	Reverse	CACACGCTGATCCAGTCAGT		
*Plzf*	Forward	TCCTCTTCCACCGCAACAG	79	XM_020097052.1
	Reverse	GCATACTCCAAAATCTGCTGAAAA		
*Nanos2*	Forward	CGGACCACTGTCGCTTCTG	159	XM_020087405.1
	Reverse	ACCGGCGTGTGTGTGCTT		

## Data Availability

The datasets used or analyzed during the current study are available from the corresponding author upon reasonable request.
